# Cyclopia: Facial deformity indicating severe holoprosencephaly with imaging findings of brain: A case report

**DOI:** 10.1016/j.radcr.2025.12.059

**Published:** 2026-02-05

**Authors:** Shiva Aryal, Bhumika Rimal, Sharma Paudel, Kundan Marasini

**Affiliations:** a Department of Radiodiagnosis and Imaging, Tribhuvan University Teaching Hospital, Institute of Medicine, Kathmandu, Nepal; b Department of Radiology, Civil Service Hospital, Kathmandu, Nepal; c Department of Radiology, Lumbini Regional Hospital, Butwal, Nepal

**Keywords:** Cyclopia, Holoprosencephaly

## Abstract

Holoprosencephaly results from incomplete separation of the cerebral hemispheres. Cyclopia is a facial manifestation of Holoprosencephaly, characterized by a midline single orbit and proboscis. Prenatal diagnosis is done by ultrasonography and can be supplemented by MRI. This case uniquely presents ultrasound and gross morphology correlation of cyclopia in a resource limited setting, and a correlation between facial features, intra-cranial anatomy, and lifespan. We report a 39-year-old gravida 3 para 2 woman with a history of alcohol consumption who presented for her first antenatal checkup at 19 weeks of gestation. Ultrasound revealed a single lateral ventricle with fused thalami, absent orbital structures, and a midline cystic protrusion (proboscis). After thorough counseling, pregnancy was terminated, and cyclopia was confirmed on gross inspection. Karyotyping and fetal echocardiography were not done due to parental preference. Alobar holoprosencephaly, the most severe subtype, is diagnosed by identification of single ventricle and associated facial anomalies. Lobar holoprosencephaly, however, requires coronal imaging to demonstrate absence of cavum septum pellucidum and fusion of frontal horns. The severity of facial anomalies correlates with underlying brain malformations. Chromosomal anomalies, maternal diabetes, infections, and teratogenic exposures like alcohol are known risk factors. Differential diagnoses include proboscis lateralis, midline encephaloceles and frontonasal dysplasia. This case highlights the prognostic significance of facial and brain anomalies and diagnostic utility of prenatal ultrasound in diagnosing holoprosencephaly underscoring the necessity of timely prenatal visits. Definitive genomic studies were not feasible, but maternal alcohol consumption can be considered as a possible risk factor.

## Introduction

Holoprosencephaly is the commonest developmental defect of the forebrain resulting from failure of the prosencephalon to divide into 2 hemispheres [1]. It is important to not confuse Holoprosencephaly with Cyclopia. Cyclopia is rather a facial manifestation associated with the most severe form of Holoprosencephaly. There is a presence of just a single eye with some duplication of the intrinsic ocular structures in Cyclopia. Cyclopia is rare, with an estimated prevalence of 1 in 100,000 live births [2]. The spectrum of Holoprosencephaly however, ranges from a single cerebral ventricle with Cyclopia in its severest form to milder forms in which affected individuals may appear normal [1]. In fact, 3 grades of increasing severity in Holoprosencephaly are described:

(a) *Alobar holoprosencephaly* (the most severe form): single ventricle, absent interhemispheric fissure; severe facial anomalies (cyclopia is a facial manifestation seen in this type).
(b) *Semi-lobar holoprosencephaly:* partial separation of hemispheres; milder facial anomalies.
(c) *Lobar holoprosencephaly:* mostly separated hemispheres; minimal facial deformities; continuity across frontal cortex may remain [3].

In Cyclopia, the top part of the skull, fronto-nasal process and facial structures may be deformed [4]. Prenatally, sonography can diagnose Holoprosencephaly and if a gross specimen is available, MRI can provide evaluation of internal structures without destroying the specimen. MRI is particularly important when autopsy or post-termination examination is limited. Risk factors for holoprosencephaly, and consequently cyclopia, include the factors listed in Table 1 [[4], [5], [6], [7], [8], [9], [10], [11], [12], [13]].

**Table 1:** Risk factors for holoprosencephaly.

Category	Examples/Details
Metabolic	Maternal diabetes
Infections during pregnancy	TORCH infections (Toxoplasmosis, Rubella, CMV, Herpes), Sexually transmitted infections
Teratogenic exposure	Aspirin, Alcohol, Lithium, Anticonvulsants, Vitamin A derivatives, Fertility drugs
Chromosomal/Genetic causes	Chromosomal abnormalities (especially Trisomy 13), Genes implicated: SHH, ZIC2, SIX3, TGIF, PTCH, GLI2, TDGF1

This case report adheres to the CARE checklist [14] and documents prenatal ultrasound-gross anatomy correlation in a resource-limited setting, with late first antenatal presentation and absent karyotyping.

## Patient information

A 39-year-old female, G3T2P0A0L2^⁎^, at 19 weeks of gestation, presented to our center for her first prenatal visit. She was married non-consanguineously. There was no prior prenatal care in current or previous pregnancies. Obstetric history included 2 full-term vaginal deliveries at home with no prior complications. She reported no preterm birth, miscarriage, or fertility treatments. Medications, prenatal vitamins, and supplements were not taken. No history of illicit drug use or smoking was reported. Past medical, surgical, and family history were unremarkable. She however consumed 1 cup(12 Fl oz) of local alcoholic beverages(≈2-3 U.S. standard drinks/day) intermittently for the past 5 years, also during her current pregnancy. Review of systems was otherwise unremarkable, with no complaints of bleeding and abdominal pain. She was advised by her friend to get a formal antenatal evaluation. After she visited our center, she was referred to us for a routine second trimester anomaly scan by the department of Ob-Gyn. The referral was routine and not prompted by specific clinical concerns.

On examination, her vital signs were within normal limits. Her BMI was 22.8 kg/m2. Her general examination revealed no pallor, jaundice, or edema. Obstetric examination was performed which revealed a soft, non-tender abdomen. The fundus was palpated to be slightly below the umbilicus. Fetal heart sounds were audible on Doppler and were 152 bpm. Systemic examinations were unremarkable.

Standard first-visit workup was done which revealed normal findings and are illustrated in Table 2 with reference ranges.

**Table 2:** Standard first-visit workup.

Test	Result	Reference range
Hemoglobin	12.3 g/dL	12-16 g/dL
Hematocrit	37 %	36%-46%
WBC count	9600 /µL	4500-11,000 /µL
Platelets	188,000 /µL	150,000-400,000 /µL
Blood type	A Positive	A/B/AB/O
Rh factor	Negative	—
Urine culture	No growth	No growth expected
HIV Ag/Ab	Negative	Negative
HBsAg	Negative	Negative
HCV antibody	Negative	Negative
RPR/VDRL	Nonreactive	Nonreactive
Sodium	142 mmol/L	135-145 mmol/L
Potassium	3.4 mmol/L	3.5-5.0 mmol/L
Chloride	102 mmol/L	98-107 mmol/L
Bicarbonate / CO₂	23 mmol/L	22-28 mmol/L
BUN	11 mg/dL	7-20 mg/dL
Creatinine	0.5 mg/dL	0.6-1.1 mg/dL
Glucose	81 mg/dL	70-99 mg/dL (fasting)
Calcium	8.8 mg/dL	8.5-10.2 mg/dL
Total Protein	5.9 g/dL	6.0-8.3 g/dL
Albumin	3.3 g/dL	3.5-5.0 g/dL

**Fig. 1:**
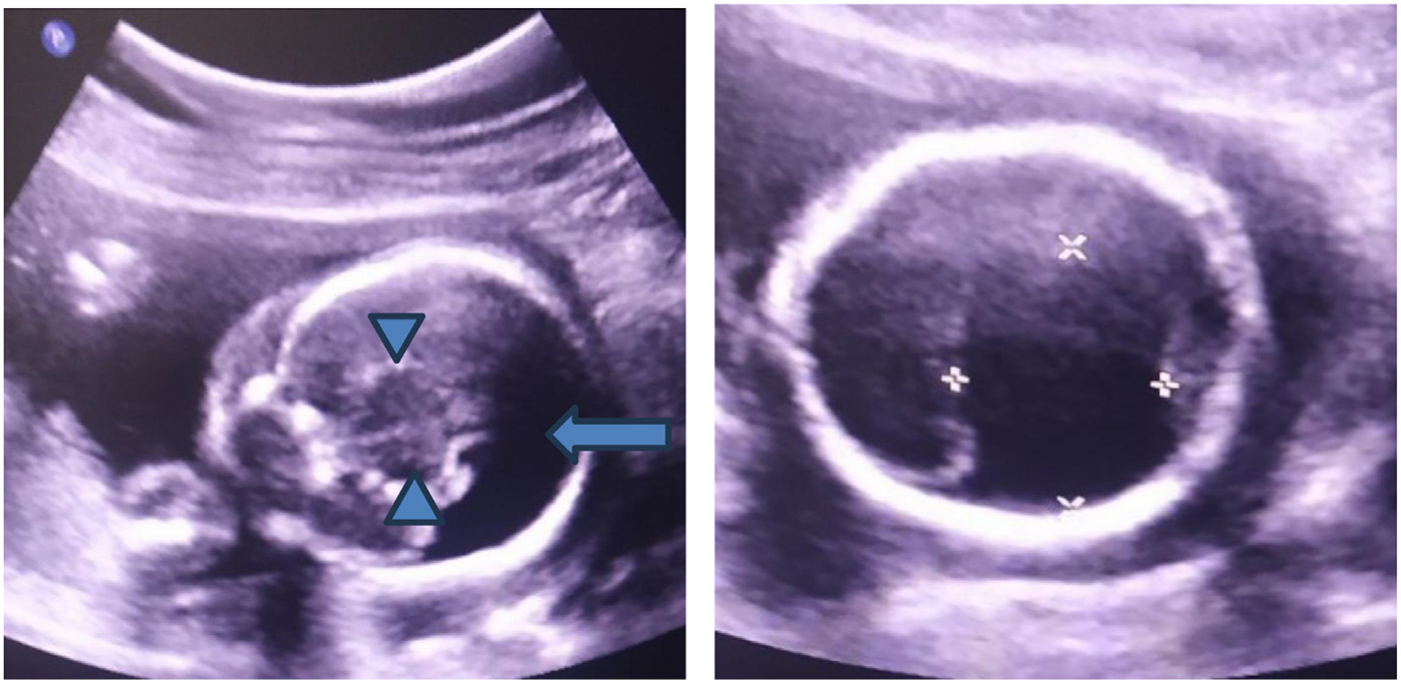
Transabdominal ultrasound of the fetal head (Philips IU22, convex probe of 1-7 MHz). (A, the left image, coronal plane) A large, single midline ventricle (blue arrow) and fused thalami (blue arrowheads) are seen. There is a complete absence of the interhemispheric fissure and falx cerebri, diagnostic of alobar holoprosencephaly. (B, the right image, axial plane) Axial view with calipers measuring the single ventricular cavity.

**Fig. 2:**
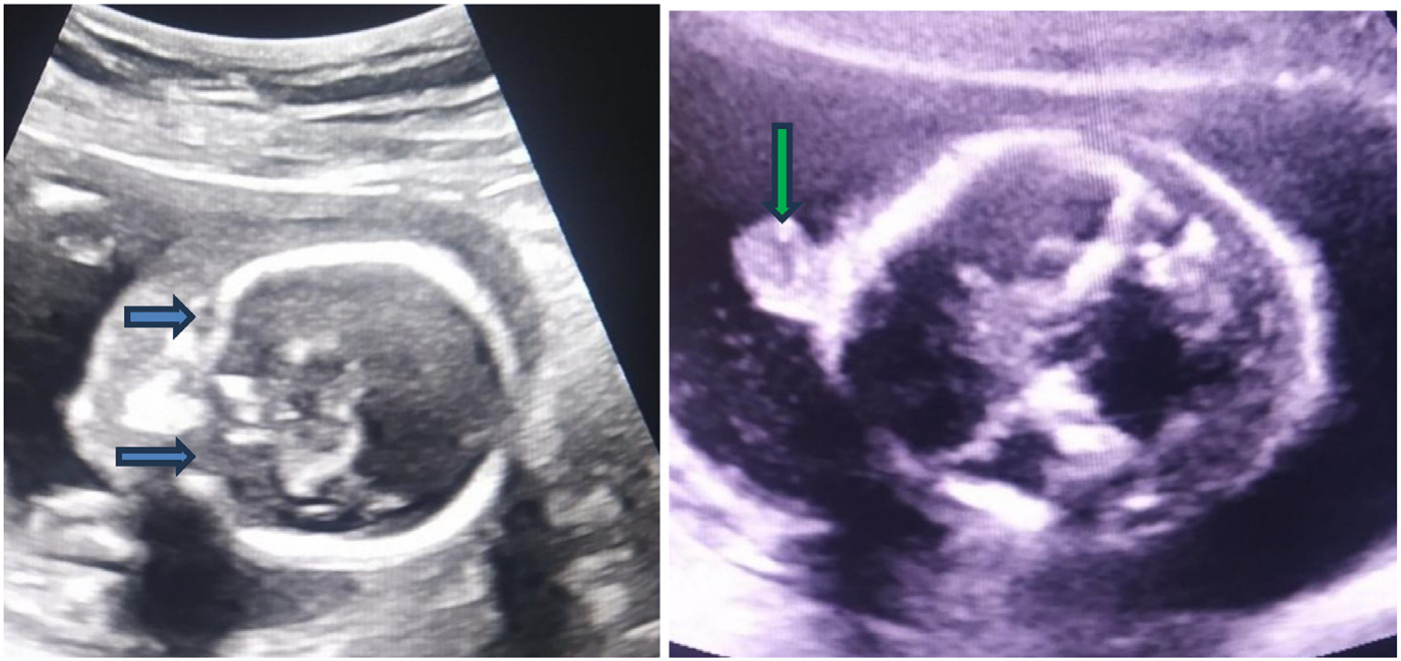
Ultrasound of the fetal face at 19 weeks gestation (Philips IU22, convex probe of 1-7 MHz). (A, the left image, coronal plane) Axial plane view showing the absence of normal orbital structures; the ocular globes are not visualized within the expected regions (blue arrows). (B, the right image, axial plane) A midline, oval, partly cystic structure is seen protruding from the forehead region (green arrow), representing a proboscis. A proboscis with absent orbits is characteristic of cyclopia associated.

**Fig. 3:**
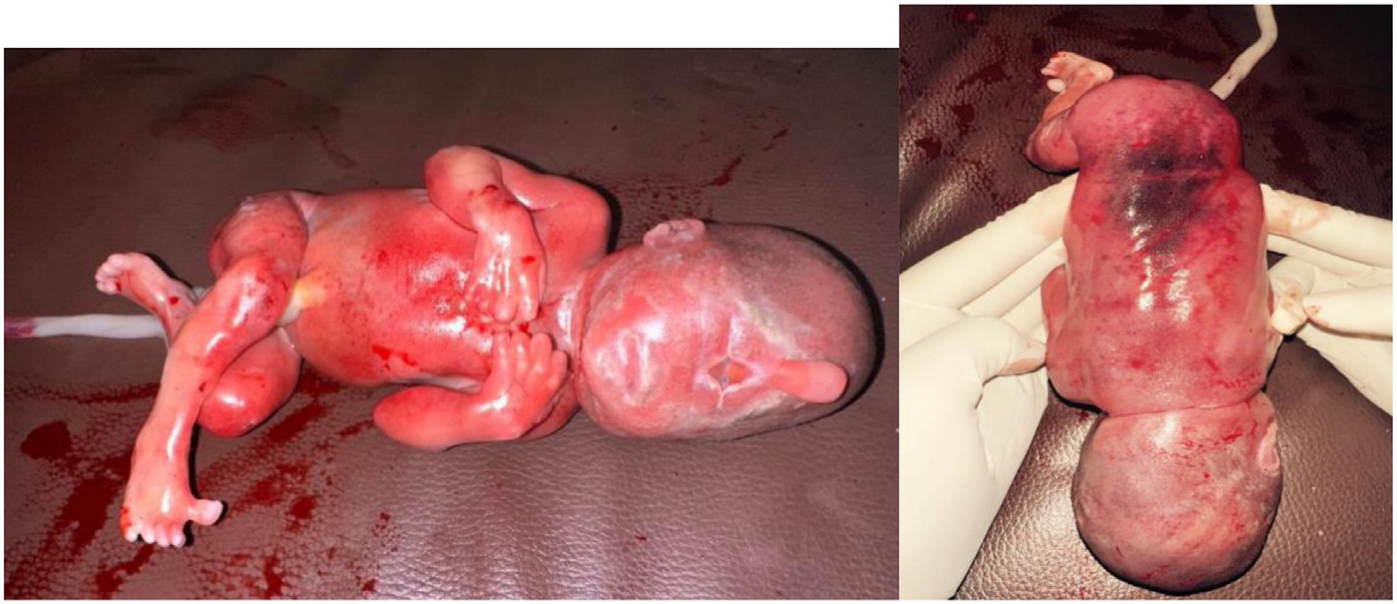
Front (A, the left image) and back side (B, the right image) photographs of terminated fetus (19 weeks of gestation) showing absent nose and both eyes with oval cystic lesion in the forehead region (A) consistent with Cyclopia corresponding with ultrasound. A bluish patch is seen in the lower back (B).

Her ultrasound findings showed a single lateral ventricle along with fused thalami and absent interhemispheric fissure, findings suggestive of alobar Holoprosencephaly (Fig. 1). The fetal face showed empty orbits and globes were not visualized. An oval, partially cystic structure protruding from the midline forehead region was also noted (Fig. 2). Additionally, a single umbilical artery was identified, and a possible cardiac abnormality was noted on sonography. Fetal echocardiography was, however, not performed due to parental refusal and structural cardiac anomalies couldn’t be excluded. Gross specimens of the fetus showed a dysmorphic face, a single median eye, absent nose, a proboscis above the eye in the forehead, micrognathia, and a purplish stain in the lower back (Fig. 3). The ultrasound findings accurately predicted the gross findings. A correlation between ultrasound and the gross findings is made on Table 3:

Published with parental consent.

**Table 3:** Correlation between ultrasound and the post-termination findings.

Ultrasound (USG) findings	Gross (post-termination) findings
Single lateral ventricle with fused thalami, suggestive of holoprosencephaly	Dysmorphic facial appearance consistent with holoprosencephaly
Absence of orbital structures with no visualization of globes	Single median eye (cyclopia)
Midline oval partly cystic structure protruding from the forehead	Proboscis above the eye with absent nose
Abnormal fetal facial morphology on ultrasound	Micrognathia

A correlation between facial anomalies and brain malformations in our case is made on Table 4.

**Table 4:** Correlation between facial anomalies and brain malformations.

Facial findings	Corresponding brain findings
Single median eye (cyclopia)	Single cerebral ventricle
Absent nose with frontal proboscis	Fused thalami
Absent orbital structures on ultrasound	Absence of interhemispheric fissure and falx cerebri
Dysmorphic midline facial structures	Alobar holoprosencephaly
Micrognathia	Severe forebrain maldevelopment

Other structures were macroscopically normal. Karyotyping and molecular testing was not done, due to lack of parental consent, hence the link to relevant mutations could not be excluded.

A multidisciplinary team comprising radiology and obstetrics confirmed the diagnosis of Cyclopia associated with alobar Holoprosencephaly. After thorough counseling regarding the prognosis and management options, the patient and her family consented to termination, which was performed by a senior obstetrician under regional anesthesia without complications. Maternal discharge was uneventful. The fetus was carefully handled and sent to the pathology department for examination, where it was confirmed to have Cyclopia with proboscis, fused orbits, and Holoprosencephaly. Gross measurements, including 14cm head circumference, and 242g body weight, were consistent with 19 weeks gestation.

A patient/family perspective could not be obtained due to the sensitive nature of this case, which is acknowledged as a limitation per CARE guidelines.

*GTPAL notation: ***G*** *= total pregnancies;* ***T*** *= term births;* ***P*** *= preterm births;* ***A*** *= abortions;* ***L*** *= living children.*

## Discussion

Early diagnosis of Holoprosencephaly - alobar type - is done by visualization of a single lateral ventricle. On the axial plane, cerebral ventricular abnormalities and facial anomalies should be identified first for the diagnosis. It might be difficult to diagnose lobar Holoprosencephaly (a less severe form) in which case a mid-coronal plane image is required to demonstrate the absence of cavum septum pellucidum and fusion of the frontal horns [15]. Fetal anomalies must be assessed sonographically in Holoprosencephaly which may range from Cyclopia to mild dysmorphisms [15]. This is important because severity of the facial anomalies correlates to the severity of brain lesions—``the face predicts the brain'' [16]. In our case, the ultrasound revealed several abnormalities. The orbits and eyeballs were not detectable, and cyst-like structures were observed in the brain, primarily on the left side. Additionally, a rounded partly cystic mass(proboscis) was noted protruding from the midline frontal area, consistent with features of Cyclopia. A differential diagnosis can be proboscis lateralis which is off-center from the vertical midline of the face and can result because of irregularity involving the nasal placode, the primary organizer of the nasal area of the midface [17]. Midline encephaloceles and frontonasal dysplasia can also present with frontal midline masses, but the orbital separation is intact. Mono-ventricle and fused thalami are not expected in them and that led to the diagnosis of alobar HPE in our case [18]. Careful assessment of intracranial anatomy therefore helps distinguish these entities from cyclopia associated with holoprosencephaly.

Limitations of autopsy can be reduced by using other imaging techniques such as MRI [19]. In our case, the ultrasound findings corroborated with gross findings, demonstrating a single median eye, frontal proboscis, and dysmorphic anterior facial structure. MRI was not done due to parental preferences which limited detailed evaluation of the forebrain and facial structures supplementing ultrasound.

Holoprosencephaly necessitates karyotyping. If karyotyping is normal, genetic counseling is the next step. If the fetus is not aneuploid (i.e. karyotyping is normal), genetic review and appropriate molecular studies are recommended [15]. Seven genes have been associated with Holoprosencephaly to this date: Sonic hedgehog (SHH), ZIC2, SIX3, TGIF, PTCH, GLI2, and TDGF1y [13]. Identification of a single umbilical artery along with a brain anomaly is also an indication for chromosomal analysis [20]. In addition, ethanol itself rather than its metabolites is strongly considered as a Holoprosencephaly inducing teratogen [21]. In our case, maternal alcohol consumption was a potential risk factor, but causality cannot be established. Genetic counseling, chromosomal studies, and molecular studies were not done due to lack of consent and/or resources, hence restricting etiologic interpretations. Another limitation of our study is that fetal echocardiography was not done due to parental refusal and thus, cardiac anomalies could not be excluded.

In contrast to the common misconception, patients with Holoprosencephaly can survive into childhood and beyond. However, when an abnormal karyotype is present, it is associated with a shorter lifespan. Just like facial deformity correlating with brain deformities, facial deformities also correlate to the lifespan in patients with normal karyotype [15]. Cyclopic infants are not found to survive beyond 1 week of age [22]. Children who survive have many problems ranging from instability of cardiovascular, respiratory, endocrine, and thermoregulatory systems, but children exhibiting mild forms may live a normal life [13]. In our case, the pregnancy was terminated by dilation and evacuation.

A list of concise teaching points is highlighted in Table 5.

**Table 5:** Teaching points.

Teaching points
Severe midline facial anomalies correlate with the severity of underlying brain malformations (“the face predicts the brain”).
A single cerebral ventricle with fused thalami on prenatal ultrasound is diagnostic of alobar holoprosencephaly.
Cyclopia represents the most severe facial manifestation of holoprosencephaly and is incompatible with long-term survival.
Early prenatal ultrasound is essential for diagnosis, counseling, and management, particularly in resource-limited settings.

## Conclusion

This presentation illustrates the importance of early imaging and routine antenatal care in detecting Cyclopia and other congenital anomalies. Cyclopia relates to the face and is the most extreme facial manifestation of alobar Holoprosencephaly. This case highlights the correlation between prenatal imaging and post-termination findings, illustrating how facial anomalies may reflect severity of brain malformations. This gives sonography or other forms of imaging substantial importance. Maternal alcohol consumption can be discouraged through better public awareness and education. This case underscores the necessity of considering both genetic and environmental factors as etiological factors. Early diagnosis enables timely counseling, informed decision making, and appropriate management of pregnancy, especially in a resource limited setting.

## CRediT authorship contribution statement

All the authors read and approved the final manuscript.

## Patient consent

Written informed consent was obtained from the patient for publication of this case report and accompanying images. A copy of the written consent is available for review by the Editor-in-Chief of this journal on request.
